# Microchimerism: Sharing Genes in Illness and in Health

**DOI:** 10.5402/2011/893819

**Published:** 2011-05-23

**Authors:** Maureen A. Knippen

**Affiliations:** Center for Biologics Evaluation and Research, US Food and Drug Administration, 5515 Security Lane, Rockville, MD 20852, USA

## Abstract

Microchimerism is defined as the presence of two genetically distinct cell populations in the same individual. It can arise from several causes including the bidirectional transfer of cells between mother and fetus during pregnancy, twin-to-twin transfer in utero, from organ transplantation, and blood transfusion. Recently, scientists have found male fetal cells from decades earlier imbedded in tissues and organs of some women with autoimmune diseases. The significance of these findings as they relate to real or potential health implications in autoimmune diseases, graft-versus-host reactions, and transfusion complications is discussed here.

## 1. Microchimerism 

**Figure 1:**
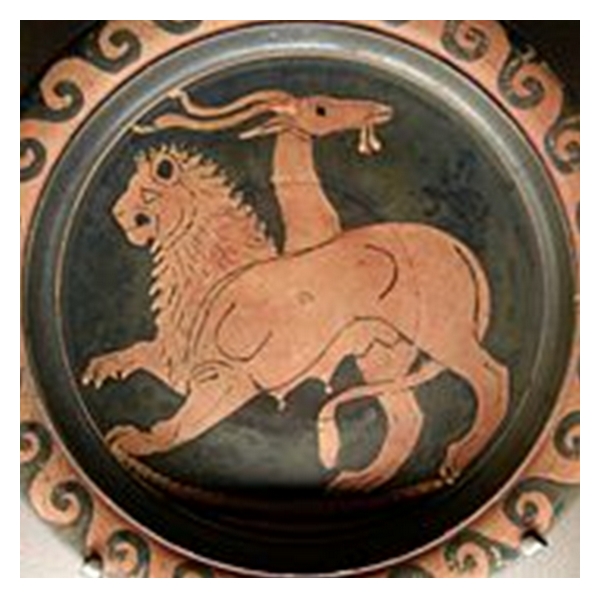
A Chimera.

Microchimerism is defined as the presence of low concentrations of two genetically distinct cell populations in the same individual. It can arise from several causes including the bidirectional transfer of cells between mother and fetus during pregnancy, twin-to-twin transfer in utero, from organ transplantation, and blood transfusion. Recently, there has been increasing interest in fetomaternal microchimerism as it relates to real or potential health implications in autoimmune diseases, graft-versus-host reactions, and transfusion complications (see Figure 1). 

The fetal and maternal cell exchange is common in pregnancy. We now know that the placenta is not an immunologically inert barrier, but rather, it allows the reciprocal transport of maternal and fetal cells in a state of mutual tolerance during gestation. To develop microchimerism, it is not necessary to continue a pregnancy and deliver an infant. Early terminations from surgical abortion can deliver up to 500,000 nucleated fetal cells into a woman's circulation [1]. Fetal microchimerism in maternal blood and tissues is typically identified using polymerase chain reaction (PCR) to identify Y-chromosome DNA sequences in the mother. Microchimerism does not require the fetus to be male, but it is easier to distinguish the Y chromosome as a biomarker. Interestingly, male microchimerism has been found in a fifth of women with no male births. This can occur in a number of ways including early miscarriage of a male embryo, a vanished male twin, male cell transfer from an older sibling through the maternal circulation to a later pregnancy, or a yet unexplored possibility, that of male DNA transferred to the woman's circulation from sexual intercourse [2]. Male fetal cells appear to have increased antigenicity in females. During pregnancy, a woman confronts an immunological challenge, because the fetus carries paternal genes, some of which are expressed on the cell surface and may provoke potent allogeneic responses. Yet, in spite of these immunologic cell differences, rejection of the fetus does not frequently occur [3]. 

### 1.1. The Role of Human Leukocyte Antigens (HLA)

The major histocompatibility complex (MHC) is the body's antiviral spyware program that resides on chromosome 6. The MHC contains genes that are involved in recognizing self from nonself and include human leukocyte antigen genes (HLA). The HLA system contains over 200 genes, 40 of which are involved with encoding leukocyte antigens [4]. HLA consists of two different classes, each with distinct functions. Class I antigens (A, B, and C) present antigens that invade cells; Class II antigens (DR, DP, and DQ) present antigens, so T-lymphocytes can determine if they should help B-cells make more antibody to a particular antigen.

The HLA genes tend to be inherited as a group, one from the mother and one from the father. The proteins encoded by HLA are the proteins on the outer part of body cells that are unique to a person. The HLA system is involved with such diverse clinical conditions as organ rejection in transplantation, death from infection in immunodeficiency, hepatic cirrhosis caused from iron overload, and autoimmune diseases [4]. This is the reason why donors are matched at major histocompatibility alleles (HLA-A,B,DR) in stem-cell transplantations. Yet, minor histocompatibility antigens such as H-Y antigen may play a bit more havoc in disease and transplant rejection than was previously thought [5]. 

### 1.2. The Y Chromosome and the H-Y Antigen

In the genetically male fetus, the Y chromosome contains a gene that codes for a protein called H-Y antigen. When H-Y antigen secretion begins, the primordial gonads become testes. Without a Y chromosome, there is no H-Y protein, and the primordial gonads become ovaries. H-Y is a minor histocompatibility antigen present on all male cells that is widely conserved in evolution [6]. It has been detected in cells from every mammalian species tested. The gene for the H-Y antigen is located on the short arm of the Y chromosome. Males develop tolerance to these self antigens, but female T cells are capable of recognizing peptides derived from H-Y proteins following transplantation. Sex-mismatched transplantations can result in major activation of the immune system even in the face of HLA-identical allografts. The risk of rejection is far greater with male solid organ grafts into female recipients [7]. 

Although the exchange of fetal maternal cells is common with all pregnancies, there appears to be increased levels of immune complexes in the serum of newborn males than in newborn females [6]. This may occur because women who have delivered male offspring can produce antibodies to H-Y proteins. In subsequent pregnancies with males, those antibodies can cross the placenta and react with circulating H-Y antigen and form immune complexes. One of the factors that increase graft-versus-host disease in allogeneic stem-cell transplantation is the use of multiparous females as donors. It has been reported that male fetal progenitor cells have been detected as long as 27 years postpartum [8]. This phenomenon, however, is not evident in all women nor is it evident in all male offspring.

 Findings from a London hospital-based study revealed that women who developed cytotoxic antibodies during their first pregnancy had significantly more male infants, suggesting that the male fetus is more strongly antigenic than the female. Secondly, they noted that in women with antibodies, the proportion of male births lessened as parity increased implying that once sensitization takes place, antibody or some related factor operates selectively in favor of the female fetus [9]. Fetomaternal red cell alloimmunization, a condition that affects one in a thousand pregnancies, involves male predominance among D+ infants who stimulate alloimmunizations in their D-mothers. The male infants have lower hemoglobin and hematocrit values and require more intrauterine, intravascular transfusions [10]. 

### 1.3. Fetal Microchimerism and Autoimmune Disease

About 80% of all people with autoimmune diseases are women. Several hypotheses have been proposed to explain the reasons for the gender difference such as hormones or stronger immune responses in women. Progressive systemic sclerosis (PSS), also known as scleroderma, is an autoimmune disease that primarily affects women in their postpartum years and bears a striking resemblance to graft-versus-host disease. Graft-versus-host disease often occurs after stem-cell transplantation. Its clinical manifestation can cause damage to the liver, gastrointestinal tract, skin, and mucosa among other bodily systems. It also resembles a number of spontaneously occurring autoimmune diseases including, Sjogren's syndrome, primary biliary cirrhosis, and system lupus erythematous. Researchers have begun looking at the association of microchimerism in certain autoimmune diseases that primarily affect women [11]. 

The first study that investigated naturally acquired microchimerism in autoimmune disease was a prospective, blinded study of fetal microchimerism in women with PSS. The study used a quantitative assay to test for male DNA in women with PSS and healthy women who had given birth to at least one son. Levels of DNA were significantly higher in women with PSS compared with healthy women. Some women with PSS who had given birth to a male infant decades earlier had results equal to the highest quartile of the assay used to test women currently pregnant with a healthy male fetus [12]. Male DNA was found 1 to 27 years after childbirth in six to eight women with PSS who had sons. Likewise, women with PSS who had given birth to at least one son had larger amounts of male DNA than healthy women and had extractable male DNA in skin lesions [13]. 

These findings ushered in a spate of studies suggesting that because most autoimmune diseases occurred in women, perhaps fetal cells played a role. Although many of these studies have noted the presence of male fetal cells in women with the disease, often times similar finding were found in control groups. For example, a study of 22 women with systemic lupus erythematosus (SLE) and a healthy control group found no difference in the amount or prevalence of male fetal cells. Male microchimeric cells were found equally in 50 percent of the women in the patient group and 50 percent in the control group. Disease activity did not appear to correlate with microchimerism. Yet, patients with a history of lupus nephritis had a higher mean number of fetal cells than patients with no such history [14]. 

Hashimoto's thyroiditis is an autoimmune disease believed to be the most common cause of primary hypothyroidism. It occurs primarily in women between the ages of 45 to 65 and has a range of symptoms including weight gain, depression, fatigue, mania, memory loss, panic attacks, and hair loss. Using thyroid gland specimens from 21 Hashimoto thyroiditis patients, 18 patients with multinodular goiter, and 17 women with normal thyroid glands (autopsy specimens), investigators found Y chromosome DNA in 8 of the 21 women with Hashimoto's disease, 1 in 18 women from the multinodular group, and none of 17 healthy thyroid glands [15].

Sjogren's syndrome is an autoimmune disease characterized by dry mouth and dry eyes that can affect other parts of the body as well. It occurs more frequently in woman over 40 years of age. Fetal microchimerism was the focus in a study of 56 women with Sjogren's syndrome, 42 of them had at least one male child. Each subject had her peripheral blood, labial salivary gland, and bronchoalveolar fluids sampled. Male DNA was shown to exist in 29% of salivary glands and 22% of lung specimens in female patients with Sjogren's. None of these subjects had a history of blood transfusion. Male chromosome PCR sequence was not detected in samples from controls. Four of the subjects who were more than 60 years old had detectable fetal cells in their peripheral blood up to 27 years postpartum [13]. One subject who tested positive for male DNA did not have a male child or a history of abortion. She did, however, have a history of blood transfusion five years before the study. It is likely the male DNA was derived from one or more transfusions in her past.

**Examples of Autoimmune Diseases Where Fetal Y-Chromosomes Were Detected Decades after Pregnancy:**

- progressive systemic sclerosis,
- Hashimoto's thyroiditis,
- systemic lupus erythematosus,
- Sjogren's syndrome.

### 1.4. Microchimerism and Blood Transfusions

Microchimerism from nonleukoreduced cellular blood products has been found to persist from months to years after transfusion. It was previously shown that in trauma patients given at least two units of nonleukoreduced red blood cells, donor leukocytes would decline markedly for the first 3 days after transfusion only to increase tenfold on day 4 followed by a secondary decline of donor cells from days 5 to 7 [16]. A subsequent study found that half of the trauma patients who received leukoreduced blood transfusions had detectable microchimeric cell populations for 2 to 3 years after transfusion [17]. Of the characteristics investigated that might explain the persistent microchimerism, only the age of the transfused unit was correlated. 

Transfusion-related acute lung injury (TRALI) is the most frequent cause of death from blood and blood-derived products in the US [18]. It is widely accepted that the transfusion-related reaction likely results from an antigen antibody reactivity between the donor's plasma and the recipient's blood cells or vice versa. The antibodies recognize targets on the recipient's white blood cells including HLA Class I and II antigens. Pregnancies are thought to be the major source of alloantibodies although prior donor transfusions may also play a role. 

In two cases presented by French physicians, TRALI reactions occurred after transfusion of products from multiparous females. The recipients developed strong alloantibodies against HLA Class I and II antigens that were present on the somatic cells of the donors' husbands and shared by the respective recipients. Typing the husbands proved useful in identifying the specificity involved in the TRALI [19]. Just as a sex-mismatched HLA-identical solid organ transplant from male to female can end in rejection, a similar interaction may be responsible for TRALI reactions with multiparous donors who have been pregnant with sons and are, therefore, microchimeric for H-Y antigen.

## 2. Discussion

The research into microchimerism, and more specifically fetomaternal microchimerism, is still in its infancy. The literature has not progressed significantly beyond a speculative role for the long-term existence of fetal cells in women with autoimmune diseases. The jury is still out as to the relationship that fetomaternal microchimerism may play in disease. Since nearly all of the studies found a significant level of normal females with microchimerism, some researchers have suggested that microchimeric cell populations that occur as a result of pregnancy may have stem-cell-like properties that can home in on damaged organs and tissues and differentiate as part of the maternal repair response [20]. 

Just as cardiovascular diseases displaced infectious illnesses with the introduction of penicillin in the 1950s, genetically derived disorders are now among the most widespread afflictions confronting contemporary society. As genetic testing becomes an integral part of patient care, nurses will be needed to provide education, counseling, and followup services. New genetic discoveries will spawn a need to rethink the nursing paradigm as it relates to education, practice, and research [20]. As knowledge of microchimerism and other genetically based illnesses expands, nurses will be confronted with how to manage the new information and challenges it will bring.
